# Potential of Black Soldier Fly Production for Pacific Small Island Developing States

**DOI:** 10.3390/ani10061038

**Published:** 2020-06-16

**Authors:** Matan Shelomi

**Affiliations:** Department of Entomology, National Taiwan University, Taipei 10617, Taiwan; mshelomi@ntu.edu.tw; Tel.: +886-02-3366-5588

**Keywords:** black soldier fly, Hermetia illucens, PSIDS, waste management

## Abstract

**Simple Summary:**

The black soldier fly is an insect of which the larvae can eat almost any organic matter, including organic waste. The larvae are edible and can be fed to livestock, fish, or even people. Around the world, black soldier fly farms and composting facilities are being established to process waste into animal feed, with economic and environmental benefits. The Pacific Small Island Developing States stand to benefit greatly from black soldier fly farming to solve multiple problems they face, due in large part to overpopulation, urbanization, and climate change. We reviewed the pressing issues in each of these nations, how black soldier fly production could help them, and which obstacles need to be cleared to make this possible.

**Abstract:**

Organic waste such as food waste and livestock manure is a serious concern in the Pacific Islands, where landfills are overflowing and illegal dumping of waste threatens the fragile ecosystems. Organic waste also attracts filth flies, some of which are vectors for pathogens that cause human disease. The black soldier fly, *Hermetia illucens*, has tremendous potential for the Pacific Islands. Capable of digesting almost any organic matter and converting it into insect biomass, black soldier flies are already being used around the world to process organic waste into larvae. The system can be adapted to large-scale municipal composting as well as small sizes for individual livestock farms or even urban households. The larvae can be fed live to fish or poultry, processed into feed comparable to fishmeal or soy meal, or even used to generate biofuel. Thus, the fly not only eliminates waste, but also can improve the sustainability of livestock production. The Pacific Small Island Developing States stand to benefit immensely from black soldier fly bioconversion facilities, used primarily as a means to compost organic waste; however, several knowledge gaps must first be addressed. We reviewed the state of black soldier flies in the Pacific and identified where their use shows the most promise. Research priorities for the field include fly surveys and bioconversion assays using Pacific crop waste.

## 1. Introduction

Insects at all life stages are rich sources of protein, fat, and many other important nutrients, and can be eaten by humans or used as feed for human food animals like fish, poultry, rabbits, and swine [1,2]. The attention given to insect farming today—by the media, by the United Nations, by entrepreneurs, and by researchers—is growing exponentially [3,4,5,6,7]. Much of this attention centers on their sustainability relative to vertebrate meat: they require orders of magnitude less feed, water, and land than mammals or birds per gram of protein, and produce similarly less waste and CO_2_. Insect meals are less environmentally destructive and often cheaper than other ingredients of animal feed, namely soy and fish meal [8]. Setting up an insect farm is cheap and maintaining one requires little labor, both attractive features for farmers in developing economies that also stand the most to benefit from insects’ nutritional status as a highly digestible, complete protein [9]. Although some insects such as mosquitoes can vector diseases like malaria or dengue fever, all such diseases either have another vertebrate primary host, or are human-specific. Since insects are so distantly related to humans, they are unlikely to be the primary hosts of zoonotic pathogens such as HIV or SARS-CoV-2, both of which have origins that can be traced to the consumption of wild animals [10,11]. (Contemporary readers may be interested to note that there are no known coronaviruses in insects or any other invertebrates [12].) Thus, their consumption and husbandry are much less likely to produce direct zoonosis of novel human diseases than vertebrate meat, although food poisoning such as botulism is still possible [13]. Insects are also sources of many different natural substances, such as chitosan, and bioactive compounds such as antimicrobial peptides that are or can be used for medical, veterinary, and agricultural purposes [14]. For many such reasons, insects are championed as a safe means to fight food insecurity and global climate change related to vertebrate consumption by replacing traditional livestock or reducing its financial and environmental costs [15,16,17].

The ability of insects to eat waste adds another benefit outside of food and feed: organic-waste management [18]. Organic waste is the single largest component of landfill waste in developed and developing nations alike, although more so in the latter [19]. This includes both pre-consumer waste—such as inedible portions of food crop plants or animals, wastes from food production and processing, and unsold food from markets—to post-consumer waste such as household and catering waste. The waste varies considerably in its composition, with examples including sugar cane bagasse, coconut husks, coffee grounds, fruit and vegetable peels, over-ripe market fruits, livestock that died of disease, offal, blood, etc. Organic-waste accumulation has serious negative impacts on the environment. It is a breeding ground for pathogenic microbes such as *Salmonella* and *Enterococcus*, and for filth flies such as corpse flies and house flies, some of which can vector these pathogens to humans and are linked to enteric disease outbreaks [20,21]. Pathogens spread by such flies range from bacteria to viruses to protozoa, and include *Escherichia coli*, *Chlamydia trachomatis* (blinding trachoma), *Clostridium difficile* (the causative agent for enterocolitis), *Cryptosporidium parvum* (Cryptosporidiosis), *Entamoeba hystolytica* (amoebic dysentery), Poliovirus (poliomyelitis), *Salmonella typhii* (typhoid fever), *Shigella* sp. (bacterial dysentery), *Treponema pallidum* (yaws), Hepatitis A and E viruses, and *Vibrio cholerae* (cholera) [21,22,23,24,25]. Some of these diseases are controllable by vaccines, but sanitation is the main or only form of prevention for others [26,27]. Pathogens, pesticides, heavy metals, and other toxins from the waste leach into the groundwater and onto agricultural land, contaminating them. Leachate also generates gases that can be explosive or toxic, or contribute to climate change [28]. On an economic level, waste management costs money and resources in collecting the waste, transporting it, and processing it through burning, composting, or, most commonly, landfilling. Unmanaged piles of waste are as unsightly as they are unsanitary, repelling tourists and the income they bring. Lastly, food waste represents a loss of resources, from those used in producing the wasted food to the nutrients locked in the waste that are not being utilized. If one adds fecal wastes, including human but typically livestock manure, then the amount of organic waste associated with food production and consumption rises and the negative environmental, public health, and economic impact of poor sanitation deepens. 

Rearing insects on waste thus solves multiple problems simultaneously. The reduction of waste means fewer disease-vectoring flies, less tourism-repelling garbage, less environmental contamination, less money spent on waste management, reduced overflowing of landfills, etc. Insects can be reared on wastes that humans and other animals cannot or will not digest, such as organic side streams from food production, catering and kitchen waste, or wastes of food-animal production itself, such as offal, manure, and corpses. The recovery of nutrients from such food wastes into edible insect biomass valorizes the waste and returns the nutrients to the food cycle, reducing the cost of food production both financially and ecologically [29]. Just as manure can be used to grow plants fed to manure-producers, so too can insects can be fed to the same organisms that produced the waste the insects were reared on, effectively closing a nutrient loop [18,30,31,32]. Insects can even be converted into biodiesel, reducing the financial and/or energetic cost of biofuel production from raw waste and helping fight global climate change [33,34,35]. The use of insects as waste reducers is expected to generate as much or more economic value than their use as food or feed, and so many countries worldwide are looking into insect-based bioconversion: processing organic waste into useful substances [29].

The utility of insects in bioconversion is not equally distributed worldwide. Different nations produce different wastes, have different laws regarding insects as food or feed, and have different infrastructures for waste management and the utilization of bioconversion products. Composting is popular in Europe, where large amounts of waste are degraded into fertilizer through microbial action, but this process is slow, requires considerable land area, and cannot process all waste types; it also requires a large farming population that will purchase the resulting compost [18]. Taiwan’s bioconversion system, where waste is collected and composted or used to feed pigs, is exemplary; however, this system cannot process all wastes and requires an extant pig farmer network, an infrastructure for biowaste collection, and a population willing to segregate their wastes [36]. Insect composting is cheaper and less technically demanding than using systems such as anaerobic digestion, and requires less land than open microbial composting, and so has considerable potential for the developing world [18,19,37]. 

Just as different nations have different needs for waste management, not all insects are suitable for all forms of waste management. Different species of insects can consume different types of organic waste (meat, manure, wood, etc.). They require different rearing conditions, with different temperature, light, and population density optima. There may be legal restrictions as to what species can be used as human food or animal feed. Lastly, if an insect is not naturally present in a certain area, then it may not be legal or advisable to import it for production, given the unknown ecological risk if it escapes. That said, one species stands out among the others. The main aim of this work was to review one particular insect, the black soldier fly (BSF), which has dominated the literature on insect bioconversion [38], and its promise for a particular region of the world, the Pacific South Island Developing States (PSIDS) [39]. These remote islands, with small land area that is further threatened by rising sea waters, have little arable land for agriculture and severe issues with waste management, such as overflowing landfills and illegal dumping of waste, on top of issues such as poverty and obesity. We concluded that BSF show outstanding promise for solving multiple problems associated with the geographic and sociological realities of the PSIDS, and delve into the remaining research tasks that must be completed before this potential can be fully realized.

## 2. Black Soldier Flies 

*Hermetia illucens*, the black soldier fly, (Diptera: Stratiomyidae) [3], is a filth-inhabiting fly [40] of which the larvae are found colonizing organic waste almost worldwide. Unlike other flies, however, they do not seem to spread diseases. Although recent studies show that, contrary to common belief, adult BSF do feed [41,42], no evidence exists to suggest that they can mechanically vector diseases the way house flies do [43]. Instead, as they are voracious feeders that eliminate large amounts of waste quickly, research shows they significantly reduce both the populations of disease-vectoring flies [44] and the numbers of enteropathogenic bacteria in the waste [30,45]. Thus BSF are valuable in efforts to close the fecal–oral route of enteric diseases mechanically vectored by other filth-inhabiting flies [21,46]. The larvae do not accumulate pesticides, pharmaceutical compounds [47,48], mycotoxins or most heavy metals [49,50], eliminating these from the waste and improving the contents of the leachate [51]. Importantly, the digestive capacity of BSF may well be the most powerful of any insect: they seem able to digest literally any organic substance soft enough to chew. Black soldier fly larvae (BSFL) can be reared on manure, feces [52], corpses, food waste, and agricultural waste, including lignocellulosic wastes that cannot be converted to biofuel and wastes high in alkaloids or other compounds that are not suitable for composting [53]. Compared to other forms of composting, BSF bioconversion produces fewer greenhouse gases and has lower global warming potential [54]. 

**Figure 1:**
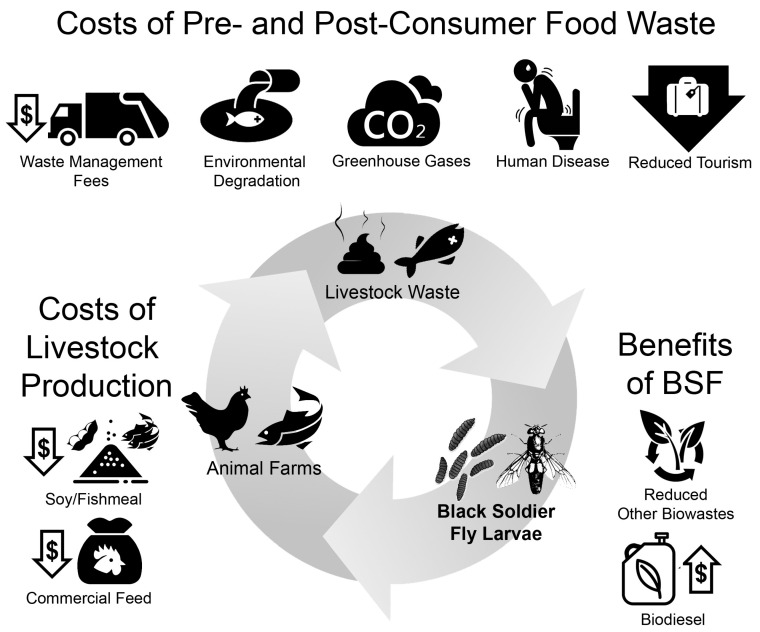
The closed-loop cycle of using black soldier flies (BSF) to process organic waste, including livestock waste itself, into livestock feed. Includes the costs of livestock production and livestock wastes, which would be reduced by BSF production, along with other benefits of BSF rearing.

Lastly, the larvae themselves are a highly desirable product. They are a biodiesel feed stock, and their use can more than double the amount of biodiesel produced from an organic waste source [33]. BSFL are also edible, especially since they do not bioaccumulate toxins or drugs, and thus can be fed to other animals whole or as a ground-up product, black soldier fly meal (BSFM) [55]. BSFM is nutritionally comparable to fishmeal or soymeal, and experiments using BSFM as a whole or partial replacement to these meals in aquaculture and poultry farming show no significant effects on the health or development of the livestock or on the quality and flavor of the meat or eggs [54,56,57,58,59,60]. Since waste from fish or poultry production, such as manure and offal, can be used to feed the same BSFL that are fed to these animals, BSF bioconversion creates a highly desirable closed-nutrient loop [61]. BSF produce little waste themselves other than some frass and shed exoskeletons, which, along with any waste they do not consume, can be valorized as a ready-to-use fertilizer product. Thus, BSF can convert waste to hygienic compost and to high-quality animal feed, reducing the financial and environmental costs of both waste management and livestock rearing (Figure 1) [27,53,54].

BSF-related products have been extensively studied, arguably more than any other insect protein, with an explosion of research in the field in recent yields [38]. As a consequence of this abundance of evidence for the safety of BSF products, several nations have legalized the use of BSF material in certain animal feeds. BSF is legal in the USA for use as feed for poultry or salmonid fish [3], and was the first insect thus approved. The European Union, which is typically cautious regarding the use of processed animal protein as animal feed, has allowed seven insects, including BSF, to be used in aquaculture feed (Regulation 2017/893) [62]. Other countries are hesitant to add laws pertaining to insects as food or feed until they are formally included in the Codex Alimentarius [63], but experimental and pilot BSF bioconversion facilities are being established in more and more countries around the world, including Nigeria, Indonesia, Taiwan, and Guam. One of the greatest success stories is AgriProtein, an originally South African company (now based in the Guildford, UK) that diverts wastes from landfills to BSF farming, making sustainable fish-oil and palm-oil substitutes and becoming one of the top three largest insect-farming businesses on earth [64]. Note that BSF is, like many insects, perfectly edible for humans: A BSF-based dairy alternative called EntoMilk™ was invented in Cape Town, South Africa that is lactose-free, and both higher in protein and more sustainable than cow’s milk [65]. 

BSF bioconversion has several advantages over other forms of waste management, besides its ability to convert almost all organic waste from cow manure to coconut shells into useful final products [66]. BSFL can be reared vertically in trays, meaning that a large-scale BSF facility that processes several tons of waste at a time does not require much space [67]. No special tools or equipment are needed, meaning that BSF rearing has low financial and technical barriers to entry, and a facility can be set up in a developing country and run with unskilled laborers [3]. A BSF bioreactor also does not need to be very large: desktop-sized BSF bioreactors exist for persons in urban areas to convert their food scraps to BSFL, though the more practical set-up is for individual farms or fisheries to process their waste and feed the larvae directly to their livestock. This would also preclude the need to transport organic waste from one place to another: waste is generated, converted, and reused in the same property. A larger, municipal-level facility can take food or even sewage waste, convert it into BSFM, and sell or give this product to farmers, thus raising money for the municipal government while also reducing costs of waste management and livestock production for farmers that would otherwise be dependent on expensive and/or imported fishmeal or soy meal. 

Based on the abundant literature on BSFL rearing [67,68,69] and use in waste processing [29,70] and livestock feeding [71], with certain strains already being bred specifically for waste valorization [72], the outlook for BSF is so positive that BSF stands to rival the honey bee and silkworm as the most economically significant domesticated insects. While much of the world can benefit from BSF, the benefits are magnified for developing economies, including, as will now be argued, the PSIDS.

## 3. Pacific Small Island Developing States 

The Pacific Small Island Developing States, or PSIDS [39], is a United-Nations-specific grouping that includes fourteen nations: the Cook Islands, Federated States of Micronesia (FSM), Fiji, Kiribati, Marshall Islands, Nauru, Niue, Palau, Papua New Guinea (PNG), Samoa, Solomon Islands, Tonga, Tuvalu, and Vanuatu. While the Cook Islands and Niue are not members of the UN, they are members of certain UN agencies and the Pacific Community, formerly the South Pacific Community (SPC). Although the term PSIDS is used, much of what is discussed in this paper applies to the other small island territories of the SPC, including but not limited to American Samoa, French Polynesia, Guam, New Caledonia, Northern Mariana Islands, Pitcairn Islands, Tokelau, and Wallis and Futuna.

These include some of the poorest countries on earth, some of which are experiencing “de-development” as the poverty situations deteriorate at rates akin to some sub-Saharan African nations [73]. Large percentages of the populations in PSIDS are engaged in subsistence agriculture [74]. Arable land is limited and at times nonexistent on these small islands and space for agriculture may be lacking, meaning that agricultural resources are valuable and environmental degradation due to waste accumulation is intolerable. The redirection of land once used for traditional food crops into growing copra for export is further exacerbating food-insecurity issues [75]. Wasted agricultural resources are a serious concern in poor countries, and expensive waste management schemes are not feasible. 

The problem of organic-waste management on PSIDS is particularly acute, even relative to other countries of similar gross domestic product (GDP). Landfilling on the PSIDS is unsustainable; in some countries, landfills are over 10 m deep in waste, and in many they are effectively at capacity, with no land available to build new ones [28]. Low-lying atoll nations have little land between the trash and the water, increasing the risks of pollution of the lagoons or seashores and of pathogen contamination of the groundwater due to landfill leachate [19]. Transporting waste around these islands is particularly expensive, as inter-island shipping is not cheap and fuel is imported at great price [76,77]. The rising ocean levels due to climate change are an added complication, causing the islands to literally sink into the sea while saltwater contaminates more and more land [78]. Rising seawater levels also can penetrate the groundwater aquifers, affecting potable water already threatened or unavailable on many islands. Organic waste represents over 40% of all solid waste in the PSIDS [19], though this can be up to 60% of solid waste per capita [28]. Customary land ownership systems reduce the ability of a municipality to establish a large waste-processing facility, so homeowners often manage all of their waste on their own property, such as by burial. Unfortunately, the majority of solid waste in PSIDS is dumped in vacant lots or even the ocean [19,79,80], with obvious negative impacts on the environment and on tourism, which several of these nations depend on as a major part of their economy. Burning piles of organic garbage is very common, but this releases harmful chemicals and greenhouse gases to the atmosphere. Mosquitoes breed in piles of garbage, such that unmanaged waste has been linked to outbreaks of dengue in the Pacific [81]. The Pacific is also an endemic area for mosquito-borne Zika and Chikungunya viruses [82]. Several of the neglected tropical diseases endemic in the Pacific are also linked to poor sanitation and filth flies, such as yaws, for which the PSIDS are a major focus and one of the last holdouts of a disease people have been trying to eradicate for decades [83], and trachoma, which can cause permanent blindness and which a 2009 survey found is surprisingly prominent throughout the PSIDS [74]. Management of solid waste, and organic waste in particular, is thus a pressing concern for almost all PSIDS [28,79]. Recycling or bioconverting waste so that it never needs to be disposed of would reduce many of these negative impacts [19]. 

Remote island nations face several other issues. Energy is particularly expensive, as oil must be shipped from afar at great cost [77]. Using waste management as a means of generating electricity would be of particular benefit to PSIDS [84]. The increasingly submerged land is becoming increasingly unable to support the population, leading in part to climate-change migrations [85]. With other issues such as loss of arable land, transitions to copra, the loss of traditional farming methods and crops that were far better suited to the island ecologies, and globalization driving demand for foreign foods [75], this has led to most PSIDS being net importers of food, if not heavily dependent on imports [86], again at high financial and carbon cost due to international shipping. While the oceans surrounding the islands have long provided resources for the people, overfishing already threatens several of these once abundant fisheries [87]. Other issues facing the PSIDS are similar to those facing developing economies worldwide, such as poverty, food insecurity, and rising malnutrition [88]. These three often come together, as lack of agricultural cultivation opportunities means less income and higher dependence on unhealthful, nutrient-poor but calorie-rich imported food, and higher rates of diabetes, obesity, and cardiovascular diseases. The top 10 most obese countries in the world by percent of the population are all PSIDS, followed by Qatar and the USA [89]. 

## 4. BSF and the PSIDS

Without minimizing the scale of the problems, many of the above issues can, to some extent, be alleviated via insect farming [17] and BSF. The PSIDS stand to gain the most from adopting BSF production, in particular as a waste-management and valorization method, to say nothing of how their weather is well suited for year-round BSF rearing and short generation times for BSF [90,91]. Several nations stand out wherein the need for BSF is highest, due to any combination of sanitation issues, enteric disease outbreaks, limited or no arable land, severe dependence on imports, and rampant unemployment, all of which are issues that BSF can help mitigate or which place BSF bioconversion as the ideal form of biowaste management. BSF farms are cheap to set up relative both to other waste-management systems and to other animal farms, creating valuable products from waste in a manner particularly suitable for low- and middle-income states [18]. Even the waste not consumed by the flies is relatively decontaminated, solving some of the chemical and pathogenic issues associated with leachates [51,92]. 

However, in effectively all of the PSIDS, the extant insect population involved in organic matter decomposition is undescribed. For some, there exist no formal records of BSF. Although the muscoid filth flies and BSF are very widely distributed, their presence is not a guarantee for the PSIDS because the distances between islands are too large for the flies to be transported naturally, some fly species are not as easily transported in human vessels, and some islands simply are not frequently visited by humans and may have never experienced an invasion event [91]. Importing a non-native insect to a country carries unknowable consequences, but BSF’s cosmopolitan distribution and lack of medical significance suggest they pose little ecological risk as an invasive species for the few parts of the world where they are not present. Nonetheless, the first step in evaluating any nation for prospective BSF farming is to search for native BSF, which would eliminate any concerns over importing a novel species and would be the main source of new flies for any newly established farm [67,93].

What follows is a listing of certain Pacific island states, their waste-management systems, the predominant issues which could potentially benefit from BSF production, and the current status of BSF therein. A summary is given in Table 1. Statistics are from the CIA World Factbook as of 2018, with specific waste-management data for every state but Niue and Tokelau from the Solid Waste Management in the Pacific Series collection of reports produced by the Asian Development Bank [28,94,95,96,97,98,99,100,101,102,103,104,105,106]. All of these countries harbor enteric diseases vectored by filth flies of the kind controllable by BSF, including Hepatitis A and typhoid, according to the International Association for Medical Assistance to Travellers (www.iamat.org).

### 4.1. Cook Islands

This self-governing country in free association with New Zealand comprises 15 islands, though nearly three quarters of its population live on Rarotonga. The population has been heavily dependent on remittances from Cook Islanders living in New Zealand or Samoa, who outnumber those still on the islands [107]. Most of the agriculture is coconuts and fruits such as papaya and citrus. Fishing and tourism are the primary sources of income, and foodstuffs a major import. The Cook Islands Ministry of Agriculture has “sector priorities” including “increasing the local production of livestock (pigs, poultry, goats)… to cater for the increased demand on the local market brought about partly by the expanded tourism industry” [108]. Land is very limited, with the modern landfill almost completely full, and sooner than expected. One reason for this is that formal organic-waste recycling does not exist, and up until recently, green waste was allowed at the landfill. Today, most of it is burned. Private operators contracted with the Ministry of Infrastructure provide waste collection to households in Rarotonga. Large resorts divert food scraps to local pig farmers, and a consortium of organic farmers does their own composting. Because literally no land exists for a new landfill, and because high dependence on tourism drives high standards for pollution control, the country has stated its desire to become “zero-waste.” BSF was recorded on Aitutaki (*Ara’ura*), the second-most-visited Cook Island located 264 km from Rarotonga, in 1960 [109]. A voucher specimen was collected in 2004 from Pukapuka [110], one of the most remote islands at 1140 km from Rarotonga, although still connected by an airport and only 757 km from Samoa. BSF are thus present in the Cook Islands, and their production would fit the national goals of boosting livestock production and reducing waste.

Note that the Cook Islands became the first “developed” country of the Pacific Islands on 10 July, 2019, according to the Organization for Economic Co-Operation and Development (OECD), and is officially a “high-income” country [111]. This is based entirely on per capita gross national income (GNI) and does not take into account the many economic and environmental vulnerabilities such island nations still face. In particular, the risk of disaster-related loss of GDP due to cyclones remains high [112]. 

### 4.2. Federated States of Micronesia (FSM)

The four Federated States of Micronesia (Chuuk, Kosrae, Pohnpei, and Yap) consist of over 600 islands spread across the Pacific north of the Equator. Heavily dependent on aid from the USA, more than 2/3 of the labor force works for the government and less than 1% in agriculture. The main economic activities are subsistence farming and fishing, but overfishing has led to depleted fish stocks. Adoption of modern lifestyles (namely the consumption of pre-prepared foods and drinks) and associated wastes that cannot be disposed of traditionally by burning, composting, or disposal into the sea has “exacerbated the problem of solid waste to the point where it is now costly to manage it” [113]. Compactor trucks for collecting waste and modern landfills were donated by Japan. Pohnpei’s old landfill had been encroaching onto mangroves and smoke from the incinerator is interfering with flights at the nearby airport. While it was partially upgraded to a “Fukuoka” style landfill [114,115] in the late 2010s, it is already overflowing. While some citizens in major towns have trash-collection services, those in other municipalities typically burn or dump waste. The last audits suggested that >42% of the waste is organic, but there is no organic-waste-recycling program. Several states are promoting household-level composting as a means to reduce waste discharge, and Chuuk state is working on installing organic-waste-disposal units made of locally available materials [113]. Recent surveys on Pohnpei found that nearly 65% of waste is generated by households, and the rest from businesses and public institutions. About 75% of waste produced by these sources is landfilled, and about 54% of landfilled waste is brought there directly by the households and businesses that generate it rather than public or private waste-collection agencies, the former heavily subsidized due to the cost of collecting greatly exceeding the fees people are willing to pay [96]. A survey of household waste sent for municipal waste management in Pohnpei found that 29.4% by weight is kitchen waste: considering that most such waste is used as livestock feed (e.g., pig slop) or firewood, this suggests that kitchen waste generation is even higher than what is collected for landfilling [116]. Fly-vectored enteric diseases are still a problem, with a cholera outbreak as recently as 2000 on the main island of Pohnpei [117]. Micronesian insect diversity has been well studied and includes BSF [118]. Thus, there should be no legal obstacles to establishing BSF facilities, with the primary goal likely being to keep organic waste out of the landfills or oceans.

### 4.3. Fiji

Fiji is a tourism, transportation (planes and ships alike), and academic hub. Fiji is one of the most developed Pacific economies, lagging behind the Cook Islands mostly due to a higher population involved in subsistence agriculture and the coups and political turmoil of the 2000s. Tourism and remittances provide much foreign funding, with bottled water and sugar being the main exports. The University of the South Pacific, the largest tertiary education and research provider across several Pacific nations, is based here. More than 80% of the solid waste from the capital, Suva, is organic waste from crops, kitchens, and markets, and another 16% is biodegradable paper and cardboard. To encourage composting, the Suva City Council limited municipal green-waste collection to monthly. An EU-funded anaerobic landfill and several private waste-collection networks exist, but illegal dumping and burning still occur. There is no large-scale organic-waste-processing facility, but home composting is encouraged and residents can buy home composting units with funding from the Global Environment Fund’s Small Grants Facility for about F$30. Fly-vectored enteric disease prevalence appears to be getting worse, not better, with typhoid becoming increasingly common [119]. 

Surprisingly, considering the relatively high educational resources there and the high likelihood of accidental import through Fiji’s well-worn international transportation routes, BSF presence in Fiji is unknown. A list from 1924 (transcribed in 2008) of all the insects in the collections of the Fiji Department of Agriculture lists three Stratiomyidae species, but no *Hermetia* [120]. A 1928 treatment of brachycerous flies in Fiji—still today the most thorough work on Fijian Stratiomyidae—listed 21 species: 18 endemic to Fiji, and the other three endemic to nearby Oceania [121,122]. BSF is thought to have reached the Pacific by the 1940s at the latest [123]; however, a 2005 arthropod survey of Fiji found the same 21 species [124]. In 2011, a novel genus of Stratiomyidae, *Vitilevumyia*, was discovered in and named after the Fijian island Viti Levu [122]. Fiji thus has very high endemism that warrants conserving, and the possibility exists that BSF may not be present on the islands. Despite a government very much eager to encourage home composting of abundant green waste, BSF may thus not be an option here, assuming that the lack of BSF is a true negative and the potential risks of importing a non-native Stratiomyidae outweigh the potential benefits.

### 4.4. Kiribati

The Republic of Kiribati consists of 33 islands scattered over 3.5 million square kilometers of ocean, with nearly half the population living in the capital of South Tarawa; one of the most densely populated and poverty-stricken places on earth [125]. It has the lowest per capita GDP of the fourteen PSIDS, with over 30% unemployment. Subsistence agriculture provides food for most families, but the sparse and widely distributed islands provide few natural resources. Human solid waste is contaminating the limited freshwater and the nation is expected to be submerged by rising ocean waters within the century should climate change continue unabated [126,127]. Residents must buy special garbage bags for non-organic waste, yet only about 38% of waste is collected, while 35% is dumped illegally into the sea or lagoons. Around 75% of generated waste by weight is organic waste, primarily fibrous garden waste from palm or *Pandanus*. Food waste is mostly fed to household pigs. A high water table and inoperable pumping systems mean that the landfills are leaching pollutants. A composting program was discussed in the early 2000s, but abandoned because of a lack of space in most households for the necessary compost heaps or banana circles [128]. A centralized composting facility at the Betio landfill is being prepared with funds from the Japan International Cooperation Agency. BSF exists here [118], although whether funding or space for BSF composting exists when other composting efforts are meeting moderate success is unknown. 

### 4.5. Marshall Islands

The Marshall Islands consist of five main islands and several atolls and smaller islands, although nearly 74% of the population lives in two cities. Coconuts and breadfruit are the primary commercial crops, although imports exceed exports here and the main source of income for the country is aid and lease payments by the USA for use of Kwajalein Atoll as a military base. Unemployment is at 36%, the highest of the PSIDS. Tourism has potential but is not as developed as in other PSIDS for which data exists. A nationalized company collects waste for landfilling in the capital city, Majuro, but several hundred households still have to transport waste to the dump themselves. The majority of people do not, instead disposing of waste in their backyards. Several dumpsites are overflowing, with toxic leachate entering the water, and problems with odor and flies. Majuro is extremely polluted, freshwater is limited, and the lagoon waters are becoming increasingly polluted from landfill leachate and other organic waste. Up to 50% of total collected waste is organic, separated at the landfill rather than by the people. Plans to generate electricity from waste are often examined, though bringing the costs per kilowatt-hour below that of diesel power is challenging. A compositing program exists, and the nationally owned Majuro Atoll Waste Company (MAWC, Majuro, Marshall Islands) sells compost it produces along with recovered metal and fuel briquettes made from recycled paper for revenue. Presently, MAWC is heavily dependent on subsidies, as tipping fees are not charged to households using the landfill, nor do they have beverage-container deposit schemes as other PSIDS do. Such fees would also promote segregation of wastes and reduce waste production at the source. BSF exists here [118], though home composting with it would require the segregation of waste at the source. Conceivably, BSF products could be added to those already sold by MAWC for revenue.

### 4.6. Nauru

Nauru is the world’s smallest republic and the 3rd smallest country after Vatican City and Monaco, at a mere 21 square kilometers of area. Its small size means that land is at a premium. Only 20% of the land has permanent crops (coconut), mostly on the shorelines which are threatened by rising ocean levels, and no arable land exists: the rest is a depleted phosphate mine, the exploitation of which ravaged the ecology [129] and the depletion of which meant unemployment at Nauru was in 2004 estimated at 90%, although as of 2011 it is estimated at 23% thanks to “secondary phosphate” mining that started in 2007. Besides phosphate, much of Nauru’s income is aid from Australia as well as revenue associated with the Nauru Regional Processing Center, an Australian immigration detention facility. The population is highly dependent on unhealthy food imports, with obesity at nearly 61%, the highest in the world. Some green waste is currently segregated to create much-needed topsoil for reclamation of the depleted mine. Waste is not collected, so individuals are required to buy bins and bring waste themselves to the landfill, which is nearing the end of its life and threatening to contaminate the groundwater with leachate. Most waste is disposed of at sea, dumped in private backyards, or burned. Sadly, Nauru lacks strong laws regulating the dumping of pollutants. The ongoing economic crisis following the depletion of the phosphate mine, lack of a dedicated waste management regulator, and lack of public understanding of waste management are further causes for concern. Regarding fly-vectored disease, Nauru has had relatively recent typhoid outbreaks [130]. Any new methods of waste management would be welcomed, but funding them is another matter entirely.

Relatively little is known about Nauru’s ecology. The first ever insect survey of the island only occurred in 2013, and it sampled from three locations far from human habitation and used methods such as pitfall traps and light traps that, while effective for moths and ants, failed to catch any flies [131]. In fact, other than mosquitoes and introduced fruit-fly pests [132], no records of any other Diptera on the island exist, which certainly cannot reflect reality. BSF’s presence is thus unknown.

### 4.7. Niue

Niue is a tiny, self-governing, coral island country in free association with New Zealand. Most agriculture is subsistence farming on family plantations, where slash and burn practices have led to reduced soil fertility. Formal paid work is almost all for the government, small industry, or the Niue Development Board. Foreign aid, remittances, and the sale of postage stamps provide much of the nation’s income. Its population is around 1600, with nearly fifteen times as many people with Niuean heritage living in New Zealand seeking economic opportunity. The first recycling facility on the island was supposed to be built by the end of 2019, but it is not for organic waste. Municipal household waste is estimated to be nearly 28% organic waste [79], most of which is burned or landfilled, though a shredder is being tested for composting and the government has implemented a pilot program to encourage composting [133]. Available entomological data are poor, decades old, did not use methods conducive to finding filth flies, and contain no reference to BSF [134,135].

### 4.8. Palau

Palau’s economy is heavily dependent on ecotourism (85% of the GDP), and environmental protectionism is a point of pride. A primary example is the tourist visa stamped into visitors’ passports, consisting of a “Palau Pledge” to protect the local environment that must be signed to enter the country. Similarly, there is widespread understanding that recycling and waste management are critical to the nation’s economic well-being. Unusual among PSIDS, illegal dumping and burning are rare here, as the government educates the populace on the importance of sanitation. Waste is segregated by households, businesses, and public institutions, and collected by the state at no charge to the user, although much of the government’s funding comes as assistance from the United States until the Compact of Free Association expires in 2024. Much waste is recycled, including 98% of aluminum cans thanks to a deposit scheme, with 10% of organic waste sent to a composting facility. The main landfill of the capital city Koror is at capacity and uncomfortably close to valuable mangrove wetlands and reefs, although a new one further inland is being developed. Lack of funding is seen as the primary issue stalling waste management. Most agriculture is subsistence-based, though, again, tourism and fishing are by far the main sources of income. BSF exists here [118,123], and environmentally conscious tour operators and hotels may be willing to adopt BSF composting, though the relative wealth of Palauans (four times higher per capita GDP than nearby Micronesians, and approaching that of Cook Islanders) means that the financial benefits of BSF are not as needed here as in more impoverished areas. On the other hand, financial obstacles to establishing such facilities are also lower, although the local government will not be the likely financier. 

### 4.9. Papua New Guinea

Although half of this large island is part of Indonesia, it is considered a PSIDS [39]. It is considerably larger (by land area) and more populated than the other PSIDS, with abundant natural resources and an extremely heterogenous population speaking 851 languages. However, difficult terrain, chronic land tenure issues, and crime problems mean that infrastructure and development are lower than they could be and poverty is widespread (around 37% below the poverty line). Mining is the most lucrative source of income, but around 85% of the labor force is in agriculture, more than any other PSIDS and despite very little land actually being classified as agricultural (more than 63% is forest). No biowaste recycling program exists, at least not in the capital of Port Moresby, though households may use food waste as animal feed. Few consumers are reached by municipal services. Illegal dumping and untidy waste storage containers are common, as are open landfills with leachate contamination of the environment and much human contact with waste. The neglected disease yaws remains a problem here [83,136]. 

Papua New Guinea is highly biodiverse with many endemic species: BSF exists here [137], among other, rarer species of *Hermetia* [138]. It is also the only PSIDS with multiple records of several different edible insects [139], although these are not commercialized. Notably, Papua New Guinea already has a vibrant insect-farming economy via butterfly ranching [140]. Demand for the bird-wing butterflies, *Ornithoptera*, including endangered species that sell for incredible prices, threatened them into extinction. In 1978, the government set up the Insect Farming and Trading Agency to promote butterfly ranching. The plants the butterflies prefer are cultivated and their forest habitats are maintained. Eggs and immature insects are protected and only a certain portion of emerging adults can be harvested for trade to insect dealers abroad, while the rest join the growing natural population. These ranched butterflies have much higher survival rates than wild butterflies, as the ranches are protected from parasitoid wasps with nets. The scheme has been incredibly successful, with hundreds of villagers in rural communities increasingly coordinating to earn income while protecting the local forests. Unique to Papua New Guinea, this government agency could greatly facilitate development of BSF-related projects.

### 4.10. Samoa

Nearly 65% of the population of this two-island nation is employed in agriculture, with fish and crops being Samoa’s main exports. Aid, remittances, tourism, and fishing provide much income. Soil erosion, deforestation, and invasive species are of great concern. Few data on waste are available. Waste on Upolu Island, which has over 70% of the population, is estimated to be 60% organic, but there is no composting program. Most households segregate their waste and compost food waste (such as taro and banana peels) themselves or feed it to their animals, such that the islands are relatively clean and free of pollution. Only about 5% of households are estimated to burn or dump waste in the ocean, and most residents are aware of the municipal waste services and are willing to learn more about waste-management issues. The landfill is relatively well maintained, and the Ministry of Natural Resources and Environment manages waste management together with the Ministry of Finance, Ministry of Health, and Ministry of Women, Community, and Social Development. Non-governmental organizations (NGOs) and international donor agencies also help. While an island-wide waste recycling program is needed, the system on Upolu at least is quite advanced compared to other PSIDS. BSF and several species of yaws-vectoring *Musca* [141,142] have been reported there [143,144]. 

### 4.11. Solomon Islands

The Solomon Islands are highly dependent on agriculture, namely fishing, and tourism. Deforestation, soil erosion, and coral bleaching are affecting these industries negatively. The rapidly growing urban population of the capital, Honiara, produces 40–50% organic waste. Only about half of the city has access to waste-collection services from the Honiara City Council’s Environmental Health Division, and the informal settlements outside the city proper get nothing. Residences, offices, businesses, and street corners are sites for garbage dumping. These piles are regularly burned, although piles sometimes grow and become mosquito breeding sites, as happened before a dengue outbreak in 2013. Even in the ten residential waste collection areas, collection schedules are unreliable. Waste is not segregated and all is landfilled or burned with regular waste. Even the waste from the central market, which is 94% food waste, is dumped in the landfill. Access to the landfill is unrestricted, meaning even less motivation for residents to segregate waste before dumping it and less chance of a quantitative waste audit. There are no municipal or commercial organic-waste-recycling or composting organizations at this time, despite the abundant organic waste. Only a local NGO, the Kastom Garden Association, promotes household-scale composting as a means to produce food organically. Residential waste collection is free, meant to be covered by property taxes despite the fact that only about 25% of property owners pay them. While multiple ministries and laws are responsible for preventing littering and maintaining cleanliness, in practice, fines for littering are not enforced. Some campaigns to promote recycling have sporadically been carried out, but promotion of composting, alerting people to the dangers of burning trash, or even publishing trash-collection routes has not been done. Financial resources, political will, and human capacity are lacking to deal with these problems. 

Unsurprisingly, given the poverty and abundant waste piles, neglected diseases like yaws remain a problem here [83,136]. BSF exists here [123,145], so the benefits of waste management with BSF are clear, yet whether the political or social will exists to implement such systems, let alone the funding, is unclear. Kastom Garden Association would likely be the first people to contact to get BSF composting started.

### 4.12. Tonga

The Kingdom of Tonga has significant agriculture covering 43.1% of its land and, with fish, comprising 2/3 of its exports. Still, much food is imported and Tonga is highly dependent on aid and remittances. Tonga is significantly affected by natural disasters, ranking third on the World Risk Index [146]. Supported by its government and Australia, Tonga updated its waste-management system following the 2005 Tonga Waste Management Act. Its population is growing, unlike some other PSIDS, and households on Tongatapu Island are estimated to produce a relatively high 200,000 tons of household waste annually. Tonga does not assess the type of waste it produces, but an audit suggested it is up to 48% organic (33% green waste from vegetation and 15% food waste). Households segregate waste, using food waste as animal feed and garden waste for compost, but there is no municipal-level composting. There is also no municipal recycling, but several private recycling companies and industries have developed around the non-organic wastes. Only 65% of urban household waste on Tongatapu is collected by the municipal waste-collection service, down to 25% in rural areas. Households can deliver the rest directly to the landfills, but the majority is burned or dumped illegally. As of 2014, the reason for this low collection rate was that only two working collection trucks existed for the entire island of over 250 square meters with over 71,000 people. Commercial and institutional waste is collected by private agencies. The landfill is appropriately sized and maintained, albeit with some areas for improvement, and is expect to last for at least another 20 years. Data suggest the waste stream is predominantly inorganic waste due to high use of organic waste by households as livestock and dog food, but recycling and waste-minimization awareness among the general populace is low.

The infrastructure of Tonga is relatively sound, so the logistics of setting up an insect farm would not be difficult, although a municipal BSF composting facility is not a priority for the underfunded waste-management services. The bigger obstacle is that BSF presence on Tonga is unknown. Data on the endemic insects of Tonga are hard to find [147] and the collection efforts to date have focused on medically significant species like mosquitoes [148]. If BSF does exist, the question of whether locals used to feeding food waste directly to pigs and poultry would be willing to use BSF to increase the nutrient quality of the waste is unknown, although commercial food producers such as breweries or fisheries might be more interested.

### 4.13. Tuvalu

The low-lying country of Tuvalu, which has a maximum elevation of just 4.6 m above sea level, is famous for being highly threatened by rising sea levels. The shrinking land area is all coral atolls, with most of the population on Funafuti atoll. Sixty percent of the land is permanent crops, namely coconut, with no arable land and effectively no agricultural labor force. Fish and copra are its main exports, with fishing, remittances, and aid as the main sources of income. Sales of .tv internet domain names brought the government some income, although not the wealth it had hoped for. Geographic isolation and increasingly limited land area pose problems for present and future waste management. Around 50% of waste is organic, according to a waste audit conducted in 2000. Households on the main island of Funafuti (only 2.4 km^2^) pay an annual fee for ad hoc waste collection, which is not enough to cover the operational budget of the municipal waste-management services. Waste is deposited in communal waste bins, with green waste intentionally left outside the bins to be collected separately, but waste is not otherwise segregated at the source. Most waste is dumped in the ocean or into “borrow pits,” which were excavated to construct Tuvalu’s airport in World War II and are now mostly filled with solid, inorganic waste. Funafuti’s official landfill is one such unlined borrow pit, long ago filled but being rehabilitated with assistance from the European Union. Taiwan’s Ministry of Foreign Affairs’ Technical Mission operates a composting facility to process agricultural waste: green waste is broken down by several chippers into a compost sold to farmers or households. This marks a successful diversion of green waste from the landfill, and NGOs and private businesses are working on recycling programs for non-organic waste. Several public-awareness programs promoting environmental stewardship and waste reduction, as well as higher penalties for illegal dumping, are working on improving recycling and composting at the source level. 

Other than crop pests and mosquitoes, data for insects on Tuvalu are scant and for filth flies effectively nonexistent [149,150,151]. Old records confirm the presence of nuisance flies and mosquitoes, but predate any possible invasion of BSF [152]. The most thorough report is an annotated biography from 1988, with no mention of *Hermetia* [153]. BSF presence is thus unknown. Given the extremely low levels of agriculture, demand for BSF as livestock feed is likely low to nonexistent, and fibrous green waste such as coconut palm fronds is not ideal for BSF. Lack of available financing and the nation’s precarious environmental position are more pressing concerns.

### 4.14. Vanuatu

Vanuatu is number one on the 2019 World Risk Index due to its exposure to cyclones, earthquakes, tsunamis, and active volcanoes [146]. Two thirds of Vanuatu’s population is involved in small-scale agriculture such as copra, cacao, coffee, fish, and beef. Agriculture is the main driver of the economy, followed by fishing, offshore banking services, and tourism. The government has sought to regulate the offshore financial centers and instead bolster agriculture, including livestock production, and tourism. Nearly 70% of Vanuatu’s population live in the Port Vila Municipality (PVM), with the land outside this area under customary ownership. Waste collection is poor everywhere, and this is stagnating the development of the tourist industry according to business, community, and government leaders alike. In the peri-urban, informal settlements, waste is mostly burned or dumped at the source. Even in tourist areas and resorts, up to half of all solid waste is burned or dumped into ravines or streams that flow to tourist destinations like waterfalls. The main dump at Port Vila, although poorly staffed, is otherwise well-managed. The municipal waste-management budget comes entirely from fees, which are seen as unacceptably high by the local residents. A 2015 waste audit found that a total of 51% of domestic waste in the PVM is vegetable and/or yard waste. Such organic wastes were 63% of waste in Luganville Municipality, and 84% in Lenakel Town Municipality, with vegetable waste 5–15 times more abundant than yard waste. There is no municipal recycling of any kind in PVM and only one private recycler focused on metal. Introducing municipal market waste composting and encouraging household composting and feeding of kitchen waste to animals are stated goals of the National Waste Management and Pollution Control Strategy, and Luganville has already established a green-waste composting facility at the market that sells compost to users [154].

Vanuatu is a biodiversity hotspot with high endemism, threatened by increasing exploitation of its natural resources [154]. The fly-vectored neglected disease yaws remains a problem here [136]. BSF exists there according to a publication [155] and a photograph on the citizen science website iNaturalist.org. The importance of waste management to tourism will be a main driver of establishing a BSF facility here. The dominant livestock of Vanuatu is beef, and, while BSF has not been studied as a cow feed outside of in vitro studies [156], its use to process, disinfect, and valorize cow manure has been studied extensively [45,46,90,157,158]. BSF as poultry feed or for more sustainable fisheries [56] would also be valued here, and a niche exists to be filled for private or public composting with or without BSF.

### 4.15. Tokelau

Tokelau is not a PSIDS *sensu stricto*, but is worth mentioning. Tokelau is a dependent territory of New Zealand moving towards free association, although both it and New Zealand consider it a separate country. Tokelau consist of three atolls with a combined area of around 10 square kilometers, and a population of less than 1500 people. Its highest point is almost 5 m above sea level, meaning that it is highly threatened by rising ocean levels and the associated loss of land and salinification of the water. Sixty percent of the land is permanent crops, namely coconut, with no arable land (coincidentally, the same as Tuvalu). It has the world’s smallest economy, dependent on coconut, postage stamp sales, foreign aid, remittances, and registration of .tk internet domain names. The per capita GDP as of 1993 (the most recent data on the CIA World Factbook) is approximately US$1000, or half that of Kiribati and fourth lowest in the world above the Central African Republic, Burundi, and the Democratic Republic of the Congo. Much waste is simply dumped on the atolls or in the sea, so solid waste management is considered a major concern of the island, for which it is seeking help from neighboring Samoa. It is of note that 100% of Tokelau’s energy is renewable, mostly solar and based on coconut oil. Entomological research on Tokelau has focused on invasive ants [159]. *Musca domestica* is there [160], but BSF presence is unknown. There is very little spare land on Tokelau, and the main substrate for BSF will likely be waste from coconuts.

## 5. Discussion

It is clear from the above list that the PSIDS are not homogenous. Big differences exist in wealth, land area, abundance of natural resources, and government competence, among other issues. Some nations have explicitly stated a desire to become zero-waste or to increase their composting rates, while others are reasonably more focused on poverty reduction. Some have populations well aware of the importance of cleaning up the environment, and some have populations already segregating and processing organic wastes at home. Some have overflowing landfills, and some have state- or city-run composting facilities. Most have significant fishing industries, many have coconut or copra farms, and all could use more funding. 

To some extent, all of the Pacific island nations, states, and territories could benefit from BSF bioconversion of organic waste. The exact extent is unknown, as those studying waste management in the Pacific have long lamented “the lack of coherent and reliable data on the types, qualities, and quantities of wastes; and what happens to those wastes” [28]. To date, only Guam, an American commonwealth, has established a pilot BSF facility [161]. Guam is a relatively well-off territory for the Pacific, with nearly 33% agricultural land and strong agricultural extension and university agricultural research systems in place. The filth flies of Guam have also been thoroughly studied [91], and a strong US military presence on Guam means filth-fly and vector-insect monitoring is performed somewhat regularly in the interest of reducing the incidence of vector-borne and enteric diseases among US forces in the area. BSF was noted there during these surveys [91]. A thorough survey of filth-inhabiting flies is a prerequisite for establishing a fly-based bioconversion facility, especially to determine whether BSF is native to the land or would need to be introduced. BSF facilities have, for example, been established with little trouble in Taiwan, as it also has well documented filth fly diversity [162] and excellent vector monitoring. 

BSF has been observed in several Pacific Island nations and territories [118,123], but not all: there are no records for Fiji, Nauru, Niue, Tokelau, Tonga, or Tuvalu, or for territories such as American Samoa (USA) [163], Norfolk Island (Australia), Tokelau (New Zealand), or Wallis and Futuna (France) [137]. For some, like Fiji, enough entomological collection efforts exist to suggest that BSF may truly be absent, in which case the ecological risk of importing a non-native species, even one as relatively harmless and cosmopolitan as BSF, may preclude the development of BSF farms. For others, entomological knowledge and records are quite poor [164]. What few insect biodiversity sampling efforts exist for the PSIDS have focused on either mosquitoes, fruit fly pests [165], or “wild” insects found in relatively undisturbed natural environments [164]. These surveys would have missed the detritivorous community associated with organic-waste-breeding insects, which will be most easily found close to human habitation, e.g., in agricultural fields feeding on livestock manure or crop waste, near latrines or toilets, or near refuse dumps and landfills. The insects in these overlooked areas are the ones for which connection to human enteric disease is highest and which have the greatest potential role in bioconversion. BSF, if present, will also be found here. The first step to bringing BSF to all the PSIDS is thus to first check whether BSF is already there through dedicated searches for them, either through hunting adults or larvae in their habitats or by using egg traps that select for BSF’s particular oviposition habit of laying eggs in crevices next to the substrate, but almost never directly on or in it [93]. 

To ensure that the people of the PSIDS benefit the most from any research undertaken there, from insect collection to eventual BSF production, the research should be local. The Papua New Guinea butterfly ranching exemplifies this: once the host plant of a butterfly is discovered by rancher–researchers, the plant’s cultivation is promoted to all members of the Insect Farming and Trading Agency. Entomological capacity building is sorely needed in some of the more impoverished areas, both in terms of facilities and equipment to the training of parataxonomists and systematists. Establishing strong entomological foundations will not only allow locals to determine for themselves (and the world) what species are present there, but also provide a population of experts who can provide support to those interested in setting up insect farms. Entomologists are also needed to apply the increasingly standardized methods of BSF research, namely resource-conversion studies [38], in order to determine the ideal parameters of a BSF facility processing local wastes (e.g. copra waste) into products for local consumption (e.g. local fisheries). The ability of insect farming, and even BSF farming, to bring economic and environmental benefits to an area is already well established through companies like AgriProtein or the work of the Aspire Food Group in Ghana [166]. NGOs and donor nations that already provide aid to many PSIDS can thus provide the initial funding to establish BSF facilities and/or entomology collections and research departments, as is being successfully done for landfills paid for by Japan in Micronesia, for example [96]. Note that there is no automatic guarantee that the benefits of science will impact rural or resource-poor farmers in a state where it is deployed, so efforts must be taken from the start to ensure that the benefits are felt by those who need them most [167].

Bioconversion research includes determining what pre-processing (grinding, dewetting or rehydrating, defatting, fermentation, etc.) and feed rates of wastes will improve the conversion process [70,168]. “Improve” in this case is specific to the needs of the community managing the BSF facility: it could mean increasing the rate of waste reduction, increasing the rate of larval biomass production, or modifying the protein:fat ratios of the larvae for the final product. Some of this research has been done for some of the wastes one would expect to predominate in the Pacific Islands. Fishing is a key part of the economy of many PSIDS, and the ability of BSF to convert fishery waste and/or to be used as fish feed has been extensively studied, as already mentioned [53], and will likely continue to be studied. BSF can eliminate waste from the production of copra and other coconut products [169]. Pre-fermentation of the coconut endosperm leads to higher protein and fat content in the larvae, making them better both nutritionally and for biodiesel [66]. More in-house research by PSIDS-based scientists on copra bioconversion will hopefully follow the establishment of BSF facilities and research programs. Other crops associated with the Pacific Islands that have been the subject of bioconversion studies in the scientific literature include pandanus [170], coffee [171], cassava [172], and oil palm [173]. Important Pacific crops for which waste bioconversion by BSF still needs to be studied at this point in time include kava, betel, noni, sugarcane, breadfruit, taro, chocolate, and citrus. The latter is important, as citrus peel has been reported to be toxic to BSF [174]. 

What scale of BSF facility is best for the PSIDS? The remoteness of many islands and their communities would justify household- or community-level BSF composting, although this will depend on whether locals are willing to put in the extra effort to produce flies relative to simply burning or burying waste. As tourism is a key component in the economies of several PSIDS, one should look into the use of medium-scale BSF facilities to process the wastes of individual resorts, reducing the costs associated with transporting waste to landfills. The downside is that resorts likely do not have any use for the BSF themselves, nor would their profit margins be greatly impacted by selling BSF products. Such medium-sized BSF bioconverters could also be used for large schools, airports, and detention facilities. A large-scale, centralized BSF composting facility is theoretically feasible, but the main limitation is the sorting, collection, and transportation of green waste to the facility and subsequent transportation of BSF products out. 

Some enterprising entrepreneurs may seek to start BSF farms less for waste removal and more for production of the larvae, if not for livestock feed, then for high-protein and low-carb food to combat unhealthy diets, or for more creative cuisine such as EntoMilk™ (Cape Town, South Africa). Legal obstacles exist in certain nations regarding the use of insects as animal feed, in large part due to the risk of prion diseases caused by feeding processed animal protein to the same animal. Present European restrictions not only limit insect feed to aquaculture, but also require the insects to have been fed non-hazardous substrates that do not contain material of animal origin. Any products made with BSF reared on post-consumer food waste would thus not be presently allowed in the EU, although these laws may change over time and with more research on the fate of prions in insects. None of the PSIDS have laws pertaining to edible insects or insects as feed as far as this author knows, which puts BSF products in a legal grey area that investors may not wish to enter. Assuming that the BSF products are legal for export, they may prove valuable. Global demand for edible insects already exceeds supply, and if Americans are willing to pay outrageous sums for bottled “Fiji Water” [175], then Pacific BSF products could similarly become another valuable source of foreign income. Remoteness and geographical fragmentation are detrimental to entrepreneurship, and the lack of economies of scale and relatively high cost of labor in several PSIDS make certain businesses uncompetitive or unfeasible here. These factors, as well as culturally-specific differences between PSIDS and Western entrepreneurs [176], mean that the BSF business plans that apply in the West may not be well-suited for the PSIDS, but, again, a growing body of research from developing nations worldwide into BSF suggests that the fly can be modified for locally appropriate uses by local users in any country. It is also critical that locals have stakes in whatever project is being undertaken, be it through awareness of the environmental benefits or the usage of fly products. Development must sustain the needs of locals without harming resources for future generations, empowering locals and providing self-reliability and self-respect rather than just profit [162].

Note that BSF is not meant to be a panacea for the ills of the PSIDS, some of which are intrinsic to the geography of small and remote islands, but others of which are primarily driven by unsustainable consumption abroad. The PSIDS face undue consequences from rising sea levels due to global climate change, despite producing few of the responsible greenhouse gases per capita relative to other nations. While boosting local entomological capacity and investing in local insect farming entrepreneurs would generate scientific, social, economic, and ethical benefits, such development is not a substitute for global-level action towards reversing or reducing humanity’s impact on the planet. BSF production could help with a great many problems, from depleted fisheries to overflowing landfills, but technologies of far greater impact and on far larger scales are needed to save sinking and cyclone-swept islands. 

## 6. Conclusions

The black soldier fly holds incredible promise as a means of converting organic waste into sustainable and cheap animal feed, addressing several problems at once. The Pacific Small Island Developing States all stand to benefit from BSF bioconversion, chiefly as a means of waste reduction, although some more than others. Research priorities that must be addressed before starting such programs include collecting or trapping efforts to check for the presence of naturalized fly populations in Fiji, Nauru, Niue, Tokelau, Tonga, and Tuvalu; bioconversion studies to determine the best way to process local waste matter for local goals; and social-science work to check the feasibility and acceptability of different BSF program scales, from municipal to institutional to household. Funding is the omnipresent obstacle for all waste-management programs, so foreign government aid and NGOs may need to provide the necessary funding to start the research and set up pilot programs. However, the local community must be active stakeholders in these initiatives in order for them to succeed and for the benefits to be felt by those who most need them.

## Figures and Tables

**Table 1:** Summary of the Pacific Small Island Developing States (PSIDS), the percentage of the land and labor force involved in agriculture, and whether or not they have black soldier fly (BSF) or municipal green-waste recycling (GRW) programs.

PSIDS	Population ^1^	Land Area ^2^	Agric. Land ^2^	Agric. Labor ^2^	BSF	GRW
Cook Islands	17,564	236 square kilometers	8.4%	29.0%	Yes	No
F.S. Micronesia	115,023	702	25.5	0.9	Yes	No
Fiji	896,445	18,274	23.3	44.0	-	Yes
Kiribati	119,449	811	12.5	15.0	Yes	No
Marshall Islands	59,190	181	33.4	11.0	Yes	Yes
Nauru	10,824	21	20.0	0	-	Yes
Niue	1626	260	19.1	-	-	No
Palau	18,094	459	10.8	1.2	Yes	Yes
PNG	8,947,024	462,840	2.6	85.0	Yes	No
Samoa	198,414	2831	12.4	65.0	Yes	No
Solomon Islands	686,884	28,896	3.9	75.0	Yes	No
Tokelau ^3^	1357	12	60.0	-	-	No
Tonga	105,695	747	43.1	27.5	-	No
Tuvalu	11,792	26	60.0	-	-	Yes
Vanuatu	307,145	12,189	15.3	65.0	Yes	No

^1^ Data from the UN World Population Prospects data for 2020. https://population.un.org/wup/, ^2^ Data from the CIA World Factbook [89], ^3^ Tokelau is not officially a PSIDS member, Agric.: Agricultural.
